# The strategic framework of tuberculosis control and prevention in the elderly: a scoping review towards End TB targets

**DOI:** 10.1186/s40249-017-0284-4

**Published:** 2017-06-01

**Authors:** Jun Li, Pui-Hong Chung, Cyrus L. K. Leung, Nobuyuki Nishikiori, Emily Y. Y. Chan, Eng-Kiong Yeoh

**Affiliations:** 1 JC School of Public Health and Primary Care, Prince of Wales Hospital, Chinese University of Hong Kong, Shatin, New Territories, Hong Kong, China; 2 World Health Organization Regional Office for the Western Pacific, Manila, Philippines

**Keywords:** Tuberculosis, Aged/older people, Strategy, Prevention and control of infectious disease, Scoping review

## Abstract

With the rapid pace of population ageing, tuberculosis (TB) in the elderly increasingly becomes a public health challenge. Despite the increasing burden and high risks for TB in the elderly, targeted strategy has not been well understood and evaluated. We undertook a scoping review to identify current TB strategies, research and policy gaps in the elderly and summarized the results within a strategic framework towards End TB targets. Databases of Embase, MEDLINE, Global health and EBM reviews were searched for original studies, review articles, and policy papers published in English between January 1990 and December 2015. Articles examining TB strategy, program, guideline or intervention in the elderly from public health perspective were included.

Nineteen articles met the inclusion criteria. Most of them were qualitative studies, issued in high- and middle-income countries and after 2000. To break the chain of TB transmission and reactivation in the elderly, infection control, interventions of avoiding delay in diagnosis and containment are essential for preventing transmission, especially in elderly institutions and aged immigrants; screening of latent TB infection and preventive therapy had effective impacts on reducing the risk of reactivation and should be used less reluctantly in older people; optimizing early case-finding with a high index of suspicion, systematic screening for prioritized high-risk groups, initial empirical and adequate follow-up treatment with close monitoring and evaluation, as well as enhanced programmatic management are fundamental pillars for active TB elimination. Evaluation of TB epidemiology, risk factors, impacts and cost-effectiveness of interventions, adopting accurate and rapid diagnostic tools, shorter and less toxic preventive therapy, are critical issues for developing strategy in the elderly towards End TB targets.

TB control strategies in the elderly were comprehensively mapped in a causal link pathway. The framework and principals identified in this study will help to evaluate and improve current program, develop targeted strategy, as well as raise more discussions on the research priority settings and policy transitions. Given the scarceness of policy and evaluated interventions, as well as the unawareness of shifting TB epidemiology and strategy especially in developing countries, the increasing need of a ready TB program for the elderly warrants further research.

**Electronic supplementary material:**

The online version of this article (doi:10.1186/s40249-017-0284-4) contains supplementary material, which is available to authorized users.

## Multilingual abstracts

Please see Additional file 1 for translations of the abstract into the five official working languages of the United Nations.

## Introduction

Tuberculosis (TB) remains one of the world’s biggest threats. It was estimated TB affected 9.6 million people and caused 1.5 million deaths in 2014 worldwide, which ranked as a leading cause of death along with human immunodeficiency virus (HIV) [1]. In order to eliminate TB, the World Health Organization (WHO) established the End TB Strategy [2], which outlined targets of 90% reduction in TB incidence and 95% reduction in TB deaths by 2035. Despite the significant progress of TB control over the last two decades, achieving the targets is a huge challenge based on current decline trend. Enhanced efforts and integrated strategies are much needed, especially for vulnerable and high-risk population for TB [2, 3].

With longer life expectancy and declining fertility rate, the global pace of population ageing is getting faster in the new century [4]. It was estimated the world’s population older than 60 years will be more than triple from 600 million in 2000 to 2 billion in 2050, gradually contributed by developing world [5]. The impacts of population ageing on TB epidemiology are complicated and may vary among countries and within countries [6]. Previous studies showed higher TB incidence and mortality in vulnerable elderly in developed world with low or intermediate TB burden, such as USA, UK, Japan, and Hong Kong [7–9]. In recent research, the same challenge had increasingly been observed in developing countries with high TB burden, such as China and India [10–12]. In 2014, the TB notification rates in people older than 65 years were higher than any other 10-years-interval groups in 13 developed and 51 developing countries/regions [1]. TB is rapidly becoming a public health challenge in the elderly worldwide.

Despite the higher TB infection rate, prevalence, incidence and mortality rate in the elderly [6], targeted strategy of control and prevention has not been well understood and examined. With the emergence of HIV-associated TB, multi-drug resistance TB (MDR-TB) and other high-risk groups, the attention appears to be diverted away from the elderly [13]. From the perspective of WHO, dozens of TB guidelines or frameworks were developed for high-risk groups including MDR-TB, TB/HIV coinfection and children [14], but few for the elderly. Previous studies in the elderly mainly focused on the TB epidemiology comparing younger people, with little effort in examining enhanced control program and evaluating targeted interventions.

Towards End TB targets, the impacts of population ageing on TB epidemiology and strategy should be drawn sufficient attentions. One study in China estimated current strategy had limited impacts on reduction of TB incidence and mortality. Additional interventions of systematic screening and preventive therapy for the elderly would enable China to nearly achieve the End TB targets [15]. Practical impacts of the elderly on the strategic targets had already been observed in Japan and Hong Kong during the past decades [6, 16]. TB strategic prioritization and transition is necessary and warrants more research based on current efforts and experience. In order to provide broad knowledge and insights for policy makers, we therefore undertook a scoping review to examine the strategy of TB control and prevention in the elderly towards End TB targets. The extent, range and nature of current research and policy activities were summarized and reported in a strategic framework for evaluating current program effectiveness, developing targeted strategy, as well as identifying policy and research gaps.

## Method

Scoping review is considered for mapping broad topics, especially where an area is complex or has not been reviewed comprehensively before. Unlike systematic reviews, scoping reviews generally address broader research questions. It can include studies of different methodological designs and do not necessarily evaluate the quality of the evidence for a meta-analysis. Given this review’s objective, we conducted a scoping review guided by Arksey and O’Malley’s methodological framework [17]. The methods were organized to the five stages laid out below, while the optional sixth stage was not carried out in this study.

### Stage 1 - Identifying the research question

The general research question this scoping review aims to answer is: What current literatures indicate about the strategy of TB control and prevention in the elderly?

### Stage 2 -Identifying relevant articles

Two authors searched articles in four databases: Three health-related (Embase, MEDLINE and Global health), and a multidisciplinary review database (EBM reviews). The search strategy was defined for each database using a combination of Mesh Terms or keywords, which comprised: tuberculosis, latent tuberculosis; aged, elderly, old adult/people; strategy, program, intervention, management, control and prevention. Keywords were adapted for each database to be consistent with their indexing. A search of reference lists of the articles included for full-text review was also conducted. We consulted one librarian and one scoping review expert for databases selection, search strategy and article selection procedures, and followed a standardized process established by the research team.

### Stage 3 -Article selection

Two authors independently screened the titles and abstracts of all articles prior to full-text review and final inclusion. For any disagreement, consultation was adopted in the research team until consensus was reached. In order to meet final inclusion criteria, articles has to: (1) be published in English between January 1990 and December 2015; (2) target exclusively in the elderly aged 60 years and older; (3) describe the strategies, programs, guidelines or interventions in TB control and prevention from public health perspective, especially aiming to reduce the incidence and mortality. Exclusion criteria are: (1) articles that are not original studies, review articles or policy papers, such as news items, letters, editorials and those in newsletters or magazines; (2) articles related to biologic, clinical and epidemiological non-interventional studies.

### Stage 4 -Charting the data

To extract data from the selected articles, an analytical framework of a data charting form was created by the database programme Microsoft Excel 2010. Charted information included author(s), year of publication, study location, study population, aim of the study, study design and method, description of strategy, key findings and conclusion. Two authors independently completed the data extraction process during review of all selected articles. Consensus was reached on the data for each study in the charting form.

### Stage 5 -Collating, summarizing and reporting results

The key strategies and interventions were identified by iterative process of data collating. A qualitative conventional content analysis was adopted to summarize and report the results [18]. The information of key findings was classified into specific categories derived from the articles rather than predefined framework. The categorization was revised with the advice of the panel of experts. General perspectives which didn’t distinguish the elderly from all age groups were not reported in this review.

## Results

**Fig. 1:**
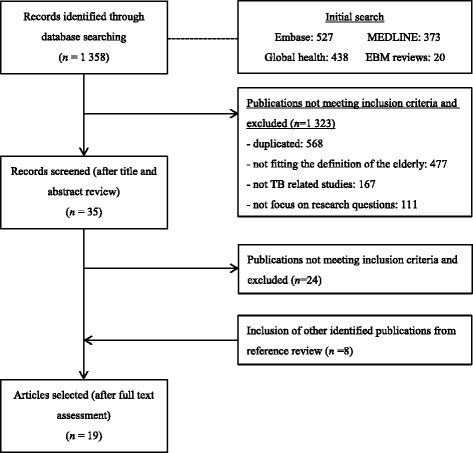
Flowchart of article search and selection

The search of all databases yielded 1 358 articles after limited to publications in English between January 1990 and December 2015 and human study. By title and abstract screening, 1 323 articles were excluded according to our inclusion and exclusion criteria. After full text assessment and reference review, 19 articles were finally retained. The article search and selection procedures are shown in Fig. 1.

### General characteristics of included studies

Studies on strategy of TB control and prevention in the elderly has most evidently emerged since 2000, with around 50% published in or later than 2005. More than half of the authors are from USA, while others are respectively from Japan, Canada, Belgium, China and South Africa. Most of articles examined from global perspective, with other three studies conducted in USA, and each two in Japan, Canada and China respectively. Given no uniform definition, the elderly was defined as people who were 65 years old and (or) older in four articles, people who were 60 years old and older in one article, or older people living in long-term care facilities in five articles; while the rest only provided vague definition. See in Table 1 and Additional file 2.

**Table 1:** General characteristic of included articles

	Number	%
Year of publication		
Before 2000	4	21%
In or after 2000	15	79%
Authors		
From USA	10	53%
From Japan, Canada and Belgium	7	37%
From China and South Africa	2	10%
Definition of elderly		
People ≥ 65 years old	4	22%
People ≥ 60 years old	1	5%
Old people living in long-term care facilities	5	26%
General concept	9	47%
Study aim		
Cost-effectiveness of case-finding strategy	4	21%
Strategy in long-term care facilities	5	26%
Others^a^	10	53%
Study method		
Qualitative review	14	74%
Quantitative analysis	5	26%

^a^Others: aim to explore the impact of interventions, general guideline, prevention and management measures, strategic priority, recommendations and policy implications

### Study aims and methods

In terms of study aims, four articles were designed to evaluate and compare the cost-effectiveness of different case-finding strategies, five articles aimed to identify the control strategy in long-term care facilities, while others aimed to explore the impact of interventions, general guideline, prevention and management measures, strategic priority, recommendations and policy implications. Most of the inclusions were narrative articles or reviews. In other four quantitative studies, three papers used decision analysis and Markov model for economic evaluation, leaving one with an individual-based computational model for impact assessment. See in Table 1 and Additional file 2.

### Strategy framework

**Fig. 2:**
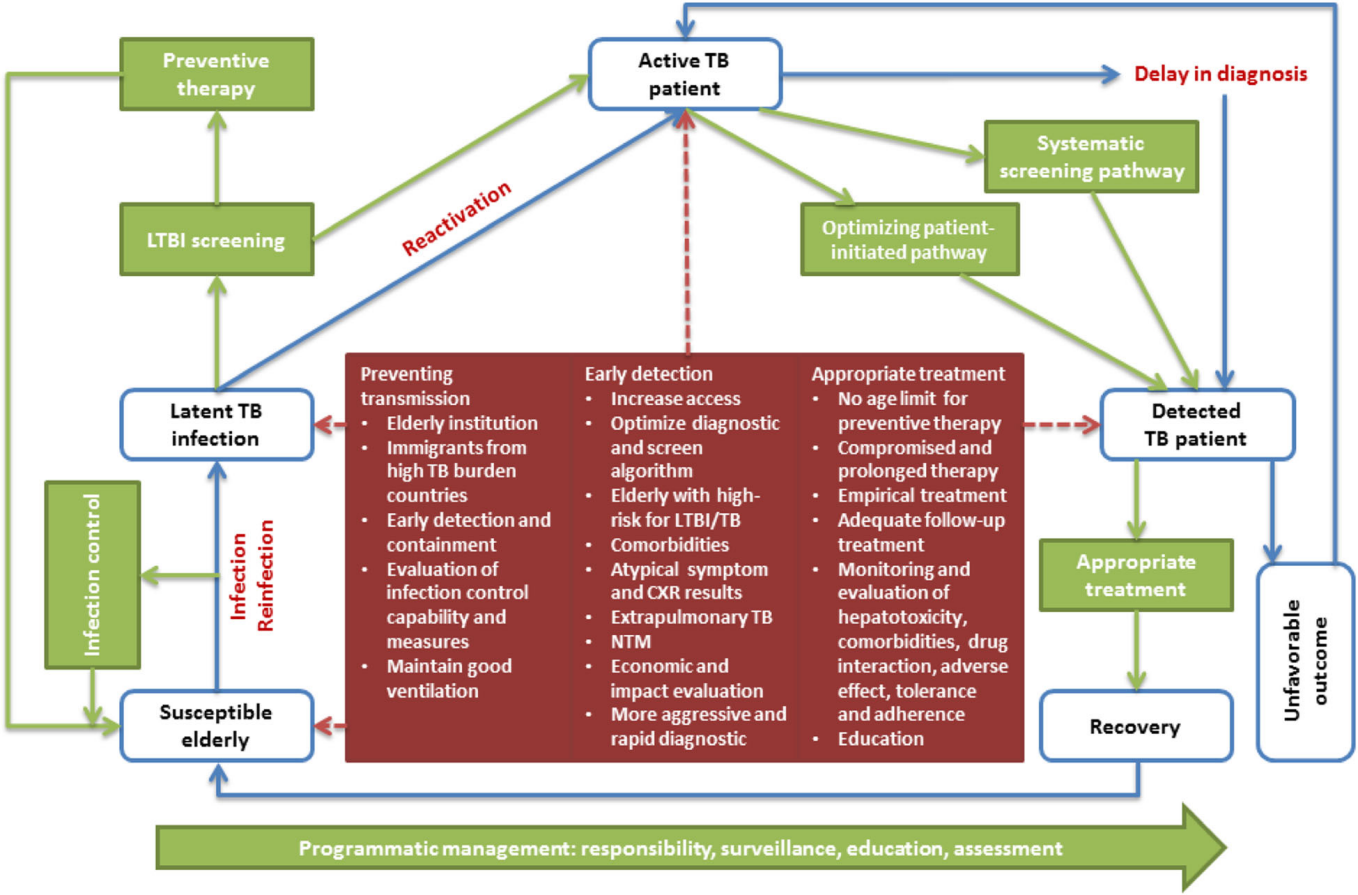
The conceptual strategic framework of TB control and prevention in the elderly

The strategies of TB control and prevention in the elderly were reported within four major categories: preventing transmission, early detection, appropriate treatment and programmatic management. The strategic framework was summarized in Table 2 and Fig. 2.

**Table 2:** Summary of strategic framework of TB control and prevention in the elderly: identified in 19 selected articles

	Strategy	Affecting factors/Strategic concerns	Suggestions/recommendations
Preventing transmission	Infection control measures [6, 13, 19–26] • Administrative actions • Engineering and environmental controls • Personal protective measures Maintaining good ventilation and avoiding overcrowding in public [6, 13, 19, 21, 23–25, 27]	Longer delay in diagnosis and treatment [6, 13, 19, 20, 22, 24, 26–28], Residents and healthcare worker in elderly institution [6, 13, 19–24, 26–28] Immigration from a high-prevalence country [13, 19, 24, 26, 28, 29]	Early diagnosis and containment [13, 19–21, 24] Evaluation of infection control measures for TB suspects and patients [21, 25, 26] • Stay in infection isolation rooms or single rooms wearing surgical masks • Transferred to a facility with appropriate isolation capacity
Early detection	Optimizing case-finding along patient-initiated pathway [15, 19, 25, 29–32] • Increasing patient access to care • Using new diagnostics • Streamlining the diagnostic pathway Systematic screening pathway in high risk groups • Screen of LTBI [6, 13, 15, 19–32] • Active case-finding of TB patients [15, 20, 21, 27, 29–31, 33] • Diagnostics and screening algorithms: TST [6, 13, 19–28, 30, 32] or IGRA [6, 26–28, 31]; CXR and bacteriological examination [15, 20, 21, 27, 29–31, 33].	High risk factors [6, 22, 23, 26, 27] • Ageing itself, male predominance, smoking, malnutrition, and BMI < 18.5 Comorbidities [6, 13, 19, 20, 22–24, 27, 28, 33] • chronic obstructive pulmonary disease, DM, lung cancer, silicosis, malignancy, liver disease, cardiovascular diseases and gastrectomy Atypical presentation [6, 13, 19, 20, 22–28, 34] • Atypical symptom: weight loss, weakness, anorexia, cognitive impairment, and dyspnoea • Atypical CXR presentation: lower lobe infiltrate, pleural effusions and extensive disease Extrapulmonary TB [6, 13, 19, 22–26, 28] and NTM [13, 19, 27, 28, 34] Economic and impact evaluation [15, 29–33] Limitation of diagnostic tools for LTBI [6, 13, 19–28, 30, 31]	A high index of suspicion and close contacts [6, 13, 19, 21–24, 26–28] Systematic screening is recommended in • Residents and healthcare workers in elderly institutions on admission and periodically [6, 13, 19–21, 24–26, 28] • Regions with high prevalence of infection [6, 13, 19, 22, 23, 27, 28] and TB disease [13, 19–21, 25, 26] • Targeted approach on high-risk groups of recent infection or reactivation [6, 13, 19, 28] • Integrating health examination [33] More aggressive diagnostic tools [6, 13, 19, 22, 23, 25, 26] Rapid diagnostic tools [22, 23, 25, 26, 28]
Appropriate treatment	Preventive treatment of LTBI • Isoniazid preventive therapy [6, 13, 15, 19–28, 30, 31] • Rifampicin preventive therapy and other alternatives [6, 13, 19, 26, 28] Treatment of TB • The therapy for elderly is not necessarily different [6, 13, 19, 24, 28], but can be compromised [13, 19, 28] or prolonged [26] • Empirical initiation of treatment for presumptive TB [13, 19, 26–28] • Adequate follow-up treatment[13, 19, 21–23, 26–28]	Hepatotoxicity for preventive therapy [6, 13, 19, 22–28, 30] Comorbidities [6, 13, 19, 20, 22–24, 27, 28, 33] Drug interaction and adverse effect [6, 13, 19, 22–24, 26, 27, 34] Poor drug tolerance [6, 26, 27] Poor treatment adherence [13, 19, 26, 27] Unfavourable treatment outcome [6, 13, 19, 27, 28]	No age limit and used less reluctantly for LTBI preventive treatment in elderly [13, 19, 26, 27, 28] Short, less toxic preventive therapy regimens [6, 15, 30] Careful pre-treatment assessment and close clinical monitoring for IPT [6, 13, 19–26, 28] Baseline and periodic laboratory testing for liver function [6, 13, 19, 22, 23, 25, 26, 28] Closer monitoring and evaluation during follow-up treatment [13, 19–21, 24, 26–28] • Evaluation of therapy compliance • Investigation of sputum conversion • Screening for adverse effects and toxicity Education [6, 20, 28]
Programmatic management	Responsibility [20, 21, 24, 25, 27] • Department of health • Primary healthcare provider Surveillance [21, 25, 27, 29] Education [19–21, 24, 25] Assessment [21, 25]	Increasing source of TB reactivation [6, 13, 15, 19, 20, 22–28] Socioeconomic determinants [6, 19, 24, 27] • Poverty, inadequate healthcare, stigma and misconception, malnutrition, unhealthy lifestyle	Awareness of changing epidemic and impact of the elderly towards End TB targets [6, 13, 15, 19, 26, 27, 29] Interventions aimed at reducing TB reactivation [6, 15, 30] Maintaining high-quality programme [30] Actions for improving socioeconomic status [6, 19, 24, 27]

#### Preventing transmission

Infection control was found as the direct and effective way of preventing transmission. Administrative actions (early identification of suspected cases, rapid isolation and appropriate treatment of infectious patients), engineering and environmental controls (negative-pressure ventilation rooms, high-efficiency particulate air filtration, and ultraviolet germicidal irradiation), and personal protective measures (masks) play essential roles in containing the infectious sources and susceptible contacts, especially in congregate setting such as elderly institutions (including care homes, residential homes, nursing homes and long-term care facilities for the elderly) [6, 13, 19–26]. For public population, maintaining good ventilation and avoiding overcrowding are also important in reducing the risk of transmission [6, 13, 19, 21, 23–25, 27]. The US Center for Disease Control (CDC) guidelines, based on a three-tier system, highlighted the prevention of the exposure of uninfected persons to active TB patients, prevention of nosocomial spread and the use of personal respiratory protection [13, 19]. In elderly institutions, an infection control committee should be set up, with qualified persons overseeing all infection control activities [21, 25, 26].

Comparing younger adults, increased risks of TB transmission were reported in the elderly due to longer delay of diagnosis and treatment [6, 13, 19, 20, 22, 24, 26–28], in institutionalized older people and healthcare workers [6, 13, 19–24, 26–28], and aged immigrants from a high-prevalence country [13, 19, 24, 26, 28, 29]. Targeted interventions were suggested to obtain an early identification and containment as soon as possible to avoid postponement of therapy and silent spread of TB infection [13, 19–21, 24]. In elderly institutions, a symptomatic patient with radiographic findings suggestive of TB disease should be placed in airborne infection isolation rooms, single rooms wearing surgical masks or transferred to a facility with appropriate isolation capacity [21, 25, 26]. Generally, patients with TB disease should not be stayed in or transferred back to elderly institutions without airborne infection isolation capability until they are no longer infectious [26]. However, it was discussed that residents with suspected or confirmed TB can remain in their usual environment without isolation precautions, under the condition of prompt diagnosis, treatment and evaluation for patients, and appropriate prevention and therapy for contacts [21, 25].

#### Early detection

There are two principal strategies for early TB detection: optimizing actions along patient-initiated pathway and systematic screening pathway. Patient-initiated pathway, also called passive case-finding strategy, is basically adopted in most countries according to DOTS (directly observed treatment, short-course) strategy. Increasing patient access to care, reducing time to treatment by using new diagnostics and/or streamlining the diagnostic pathway are essential interventions to optimize current strategy [15, 19, 25, 29–32]. Systematic screening pathway comprises screening for latent TB infection (LTBI) [6, 13, 15, 19–32] and screening for TB patients [15, 20, 21, 27, 29–31, 33]. The latter is also called active case-finding (ACF). Screening for LTBI relies on the measurement of cellular responses to TB antigens, either by tuberculin skin test (TST) [6, 13, 19–28, 30, 32] or Interferon-Gamma Release Assay (IGRA) [6, 26–28, 31]. For ACF, chest X-ray (CXR) is mainly adopted such as in contact investigation or in combination with annual health screening program. Bacteriological examination will be provided for those with TST/IGRA positive and/or abnormal results of CXR.

Diagnostics of screening LTBI/TB in the elderly is complex and widely discussed. TST remains the gold standard test and the diagnostic intervention of choice for screening LTBI for many years [6, 22, 25, 26, 28]. In the elderly, owing to few opportunity in receiving Bacillus Calmette-Guerin (BCG) vaccination in their childhood and various immunologic response, multiple cut-offs are therefore recommended to give the best predictive values under different clinical and epidemiologic situations [6, 13, 19]. Factors such as separate test-reading visit, potential boosting of response on serial testing ascribed to immune-compromised, as well as cross-reactivity with the BCG and nontuberculous mycobacteria (NTM), affect its field application with decreased sensitivity and specificity in the elderly [6, 13, 19–28, 30, 31]. IGRAs are new alternatives to TST with at least equivalent sensitivity and higher specificity [6, 31]. In spite of operational advantages (one step test, quick result), less affected by advance age, BCG status and booster phenomenon, large-scale application of IGRAs may be limited due to the need of delivering fresh blood sample, lengthy laboratory processes and higher costs [6]. Given the limitations of LTBI tests, the prevalence of background infection among different places and settings should be taken into account [6, 13, 19, 22, 23, 27, 28]. Better cost-effectiveness could be achieved using a more targeted approach, focusing on risk groups such as the elderly with a higher risk of recent infection or reactivation [6, 13, 19, 28].

Evaluating the cost-effectiveness and impacts of systematic screening were mainly concerned under different circumstance. Based on three studies in particular long-term care facility and BCG-vaccinated elderly, no screening strategy offered the greatest cost savings [31]; screening with TST was more cost-effective than CXR screening or passive strategy [30, 32]; IGRAs may become more cost-effective when its sensitivity was over 0.89 and TB prevalence was higher [31]; CXR screening was less cost-effective than no screening for BCG-vaccinated elderly [31]. It was also noted that for newly admitted elderly in long-term care facilities, although the health benefits of screening were significant [13, 19, 32], strategies might not be cost-effective in a low-burden setting [30]. Identifying the elderly with highest reactivation risk would improve the cost-effectiveness of screening [30]. Economic evaluation was also considered in national TB control strategies. In Japan, the BCG immunization and Mass Miniature Radiography (MMR) in the young population were suggested to be abandoned because of cost-ineffectiveness [29]. Instead, the elderly should be strategically prioritized, by developing both active and passive case-finding through public and community health services [29]. In China, it was estimated that screening for LTBI and ACF in the elderly would result in a decline in TB incidence and mortality of 48% (34–64%) and 58% (40–72%) in the next 20 years [15]. ACF in health examinations for TB was effective for the elderly in rural areas, especially among elderly diabetes mellitus (DM) patients with TB symptoms [33].

Specific characteristics which would impact early detection in the elderly should be altered. (a) High-risk factors. Ageing itself, male predominance, smoking, malnutrition, and BMI < 18.5 are the risk factors for developing TB in the elderly [6, 22, 23, 26, 27]. (b) Comorbidities. Elderly TB patients have more risks for chronic obstructive pulmonary disease, DM, liver disease, malignancy, cardiovascular diseases and gastrectomy owing to decreased immunocompetence [6, 13, 19, 20, 22–24, 27, 28, 33]. (c) Atypical presentation [6, 13, 19, 20, 22–28, 34]. Fever, productive cough, night sweats, and haemoptysis are less frequent in older patients, while weight loss, weakness, anorexia, cognitive impairment, and dyspnoea are more common. Radiographic findings such as cavity formation and lesions in the upper lung area are rare in elderly TB, while lower lobe infiltrate, pleural effusions and extensive disease are more common. (d) Extrapulmonary TB (miliary, pleural, lymph node, TB meningitis, skeletal, genitourinary and craniospinal TB) [6, 13, 19, 22–26, 28] and NTM [13, 19, 27, 28, 34] are observed increasingly with advancing age.

Therefore, a high index of suspicion and close contacts should continue to be alerted and required to detect the atypically presenting disease in the elderly [6, 13, 19, 21–24, 26–28]. Regarding screening all residents and healthcare workers in elderly institutions [6, 13, 19–21, 24–26, 28], two-step TST method is recommended to firstly establish a baseline and prevent incorrect identification of conversion with subsequent periodic screening [6, 20, 21, 24, 26, 28]. All persons with a positive reaction should receive a chest radiograph to identify current or past tuberculous disease [25, 35]. Periodical test should be performed for residents and healthcare workers closely contacting TB patients, with arising suspected TB symptoms, developing TB disease or TST conversions, or in regions with a significant prevalence of TB disease [13, 19–21, 25, 26]. In addition, screening for LTBI/TB is also recommended to high-risk groups including those who are immunosuppressed because of disease (HIV infection) or medications (corticosteroids), recent close contacts of infectious TB patients, and those with abnormal chest radiographs suggestive of prior TB [28]. However, another study showed close contacts may not necessarily pose a greater risk of TB among the elderly [33]. Apart from standardized diagnostic tools, more aggressive diagnostic intervention like fiberoptic bronchoscopy with peripheral biopsy specimens [6, 13, 19, 22, 23, 25, 26] and rapid diagnostic tools of TB [22, 23, 25, 26, 28] should be considered for the elderly.

#### Appropriate treatment

Early LTBI/TB detection may become useless without corresponding interventions for preventing TB progression from LTBI, as well as preventing new transmission and unfavourable treatment outcomes in TB patients. Preventive treatment in the elderly is recognized as the most effective single intervention which can directly reduce the size of the latent reservoir [15]. Isoniazid preventive therapy (IPT) is the preferred therapy [6, 13, 15, 19–28, 30, 31], with the regime ranged from 6 months to 9 month, and 12 months advised for HIV-infected patients [6, 13, 19, 20, 22, 25]. Other studies indicated 9 months was preferred to the 6 or 12 months regimen regardless of HIV status [15, 26, 28]. The key limitation of IPT is the higher risk of hepatotoxicity increased with age [6, 13, 19, 22–28, 30]. However, recent guidelines recommended that only treating LTBI in persons younger than 35 years old should be abandoned. If it is believed the benefit exceed the risk of side effects and towards eliminating TB worldwide, treatment of LTBI should be no age limit and should be used less reluctantly in the elderly [13, 19, 26–28]. The 4–6 months Rifampicin preventive treatment and other alternative therapies for treatment of LTBI need further examination on their tolerance, toxicity and efficacy [6, 13, 19, 26, 28].

In general, the recommendations for the treatment of TB in the elderly are not necessarily different from those in younger adults [6, 13, 19, 24, 26, 28], but could be compromised due to the frailty, presence of concomitant diseases and adverse drug reaction [13, 19, 28]. The elderly may require longer duration of therapy, generally 9 months rather than standardised 6 months [26]. Empirical initiation of treatment for the elderly is recommended in TB suspects due to diagnosis difficulty, poor treatment outcome and consequently high risk for transmission [13, 19, 26–28]. Adequate follow-up treatment, as one key element of DOTS strategy, is also essential to elderly TB patients [13, 19, 21–23, 26–28].

Special attentions for treatment in the elderly should be paid to prevent, detect, and manage more risks of: (a) hepatotoxicity for IPT [6, 13, 19, 22–28, 30]; (b) comorbidities [6, 13, 19, 20, 22–24, 27, 28, 33]; (c) drug interaction and adverse effect [6, 13, 19, 22–24, 26, 27, 34]; (d) poor drug tolerance [6, 26, 27]; (e) poor treatment adherence [13, 19, 26, 27] and (f) unfavourable treatment outcome [6, 13, 19, 27, 28]. Accordingly, careful pre-treatment assessment and close clinical monitoring for IPT [6, 13, 19–26, 28], baseline and periodic (monthly or biweekly) laboratory testing for liver function [6, 13, 19, 22, 23, 25, 26, 28], closer monitoring and evaluation during follow-up treatment [13, 19–21, 24, 26–28] and education [6, 20, 28] are crucial to minimize the above potential risks. Routine biochemical monitoring is strongly recommended for selected elderly residents with advanced age, multiple comorbidities, an inability to report symptoms reliably, abnormal results on baseline liver function tests, and concomitant use of other potentially hepatotoxic medications [26]. Follow-up examinations should include evaluation of compliance with therapy, investigation of sputum conversion and screening for adverse effects and toxicity of the regimens [13, 26].

#### Programmatic management

Programmatic management is designed to effectively implement the strategic plan through following key measures. In a uniform national surveillance system, all elderly TB patients, infected residents and staff in healthcare facility and community should be identified and reported promptly either in active or passive case-finding strategies [21, 25, 27, 29]. Education of targeted TB knowledge should be provided for information and imparting skills to healthcare workers, patients, families, visitors, and employees so that they can all understand and be engaged into TB control programme [19–21, 24, 25]. Assessments are needed to monitor and evaluate the activities in line with TB control programme and the responsibilities [21, 25]. Responsibility of department of health and primary healthcare provider should be strengthened [20, 21, 24, 25, 27]. The health departments should assist in developing and updating policies, procedures, and record systems, providing epidemiological and management assistance, consultation, program training, and assessment [20, 21, 25]. The primary healthcare providers play a pivotal role in educating the individual, encouraging follow-up, and directing to support system [20, 24, 27].

The higher burden of infection in the older cohorts, higher disease risk with age, as well as higher chance of remaining latent for many years before reactivation are the key obstacles to the elimination of TB, since endogenous reactivation of remote infection in the elderly is becoming the main sources of TB morbidity and mortality [6, 13, 15, 19, 20, 22–28]. Therefore, shorter, less toxic preventive therapy regimens and interventions aimed at reducing reactivation from the latent reservoir seem more effective to rapidly reduce TB burden [6, 15, 30], as shown in the modelling study in China [15]. Until these become available, closely monitoring the changing demographics in TB patients, education and contact investigation need to be maintained with the assistance of public health or the TB programme [30]. Towards socio-economic determinants for TB, poverty reduction, provision of adequate healthcare, elimination of stigma and misconception, optimal nutrition, careful control of high risk factors by improving general health status and managing comorbidities should also be taken into account [6, 19, 24, 27].

## Discussion

This scoping review summarized articles aiming to examine strategy of TB control and prevention in the elderly. The key results demonstrated what strategies and interventions were being examined, why and how they were being conducted, as well as policy implications with affecting factors, strategic concerns, suggestions and recommendations. The objective of TB control strategy in the elderly is to eliminate TB by breaking the chain of transmission and reactivation [19]. In this study, it could be achieved within the strategic framework: preventing transmission among susceptible elderly, screening and preventive treatment of high-risk groups with LTBI, rapid detection and effective treatment with close monitoring of TB patients, and programmatic management for integrating all available resources and interventions.

The challenge of TB in the elderly had been acknowledged in high-income countries, but less considered in developing world, as shown in the results. However, it is the time to take notice. In China, the proportion of people 60 years or above was 13.3% among overall population, but as high as 48.8% among TB patients in 2010 [36, 37]. The TB prevalence in elderly was more than three-fold higher than the one in younger generation [11]. The Disability-Adjusted Life Years lost due to TB among the elderly was estimated higher in South, East and Central Asia, Latin America and Caribbean than the global level [10]. In addition to the high TB burden, a large amount of elderly TB patients might be undiagnosed or delayed in diagnosis more likely in low and middle-income countries. From one study in Cambodia, the elderly patients accounted for 23.2% in a community-based ACF project, nearly double the proportion in this age group by passive case-finding [38]. Similar results were also observed in South India and Eastern Nepal [39, 40]. Even in Africa countries, increasing TB burden was found in the elderly, shifting from young HIV-infected to older HIV-uninfected people, who may not easily access to healthcare comparing younger population [10]. Therefore, both developed and developing countries should ensure the TB programmes are ready for the demographic shift in TB patients.

Screening and preventive treatment for LTBI is conditional among various high-risk groups for TB. From current WHO guideline on management of LTBI [41], although age limits had been removed, the elderly is still not prioritized mainly due to the higher risk of hepatotoxicity by IPT, as shown in the results. In previous studies, higher rates of isoniazid intolerance and hepatitis were observed in patients older than 50 years, and fatalities occasionally occurred [42–44]. However, in most cases of serious toxicity, the patient either was not monitored for clinical or biochemical toxicity, continued to take the drug without specific guidance, or had complicating underlying liver disease or alcohol abuse. Abnormal liver function was found with more impacts than age on the development of isoniazid-induced hepatotoxicity [45]. In a systematic review of age-related risk of hepatotoxicity by treating LTBI in 2010, the overall rates of hepatotoxicity were low; associated hospitalisation or mortality was extremely uncommon [46]. The use of IPT was concluded as safe in older patients with close clinical or biochemical monitoring and education [46], which had also been documented by recent studies [45, 47].

The reason of increasing need for LTBI interventions in the elderly is based on the fact ageing TB epidemic largely postpones the End TB targets. There is little doubt that DOTS strategy can effectively contain the transmission and reduce diseases developing from progressive primary infection or exogenous reinfection in the community. However, TB developing from endogenous reactivation is less affected. Hence, in places successfully implementing DOTS for a long time, the TB epidemic would gradually evolve into cases developed mainly from reactivation, with fewer cases from infection and reinfection [48]. Unfortunately, the elderly is the largest reservoir of LTBI and has higher risk for reactivation. The pool of infected persons, who are getting older with residual effects in earlier life, cancels out the continuing success of battle against TB [16, 49]. This could mainly explain the high proportion of elderly TB patients in low incidence countries and stagnant endemic in intermediate TB burden regions [7, 50]. Recently, it could also be increasingly observed in developing countries with high TB burden. In China, given a scaling up of DOTS strategy since 1990, it is not surprising the prevalence of all pulmonary TB declined much slower from 2000 to 2010 than the previous decade [11]. Targeted interventions for LTBI should be considered to be paramount if we are to eliminate TB worldwide.

Early diagnosis and prompt treatment for all persons of all ages are consistently essential. Likewise, systematic screening of the elderly and those in residential institutions for active TB are conditionally recommended, on basis of assessing specific epidemiological, social and health-system situations, the relevance and cost-effectiveness [51–53]. In addition, interventions should be tailor-made to their specific characteristics. First, male, smokers, malnourished, migrants, people with comorbidities and other risk factors for TB in the elderly should be altered in surveillance and program design [54, 55]. For instance, DM was recognized as a great contribution to TB in the elderly [56]. Assuming 25% elder adults with DM [57, 58], targeted systematic screening would achieve more yields, cost-effectiveness and feasibility particularly in elderly institutions or integrated into an existed health examination program. Second, from health providers’ perspective, a high index of suspicion and further bacteriological examination in the elderly should be highlighted in line with the atypical presentation and higher risks for extrapulmonary TB and NTM [54, 59, 60]. There is also a practical need for more accurate and rapid diagnostic tools. Third, higher mortality and poor treatment outcome in the elderly is associated with delay in treatment, comorbidity and poor treatment adherence [55]. One study suggested that very old patients with TB had higher mortality; but if diagnosed early and treated adequately, they did not show greater mortality [61]. Hence, high awareness, timely intervention and close monitoring of treatment are highly recommended [62].

Attention should also be paid to the elderly institutions due to the higher LTBI/TB prevalence in elderly congregate settings, which were widely observed in low-incidence countries like United States [8, 21, 63], and in intermediate TB burden regions like Hong Kong [64–66]. With appropriate infection control measures, the risk of infection is only 0.6–1.2% with no cases of active disease, while the infection risk can reach 7.7% without measures [67]. Therefore, infection control measures were further recommended by CDC to be operated in all health-care settings rather than only in long term care facilities [68]. Regarding other potential interventions, BCG and MDR-TB were found with fewer impacts on the elderly than young people, presumably due to their infection time prior to available BCG and effective chemotherapy [13, 19, 22, 24, 25, 27, 28]. However, antimicrobial susceptibility test was still recommended to identically conduct in the elderly for clinical management in regions of increasing drug resistance due to higher risk of drug toxicity and intolerance [26]. Among people living with HIV, few specific control strategies were proposed for the elderly. With the ageing of HIV epidemic, further research and policy guidance are expected to bridge the gaps.

## Research gaps

In this review, few official TB control strategy, framework or guideline for the elderly were identified and adopted in global, regional or national TB programme. Comparing to the increasing needs, more efforts and research should be done to address this neglected challenge, especially in the developing countries. Furthermore, little evidence of evaluated interventions, such as studies adopting randomized control trial, was found in this paper. The effects of proposed interventions should be further examined by practical and evidence-informed evaluation. For instance, although TB systematic screening has been studied in aspects of methodology, algorithms and in different high-risk populations, the direct evidence remains weak for the impact of screening on health outcomes and TB transmission when compared with passive case-finding alone [51, 52, 69]. Some new diagnostic tools, like Xpert MTB/RIF, have been evaluated recently in many high-risk groups other than in the elderly [69–71]. The evaluation of cost-effectiveness, feasibility and acceptability under specific circumstances also lacks independent study design in the elderly. Third, towards End TB targets, the limitation of diagnostic and therapy of LTBI/TB must be overcome. It requires intensified innovation from fundamental research for improved diagnostics, medicines and even vaccines, to operational and health systems research to improve current programmatic performance and introduce novel interventions with new tools [2]. Finally, other affecting factors and potential interventions for the elderly should not be ignored. Poverty, health inequality, stigma, socioeconomic disadvantage, illiteracy and low awareness might be largely associated with the elderly and TB. Accordingly, interventions of providing more accessible healthcare service, psychosocial and financial support will positively contribute to the program.

## Limitations

This is one pioneering study from public health perspective which responds to the neglected TB challenge with global ageing of population. One aim of this scoping review is to identify the strategies within the framework as comprehensive as possible. However, some regional TB policy or practical experiences might not be included since they were only published in domestic languages rather than English or even unpublished. Moreover, owing to the scarceness of available policy resources and evaluated interventions, the unawareness and little evidence in developing countries, as well as the wide range of study designs included in the scoping review, the direct extrapolation and application of the experience within the framework should be very careful. More specific strategies under different circumstances in line with regional TB epidemiology, socioeconomic and health-system situations warrant further research. In addition to the public health interventions for breaking the chain of TB transmission and reactivation, the development in biologic and clinic medical science, patient support and care responding to the socio-economic determinants should also be taken into account, although they are not the main focus in this study.

## Conclusions

This scoping review systematically examined the strategy of TB control and prevention in the elderly through literature resources. The framework used in this study helped to characterize the strategies with specific concerns in a causal link pathway: preventing transmission among susceptible elderly, screening and preventive treatment of high-risk groups with LTBI, rapid detection and effective treatment with close monitoring of TB patients, and programmatic management for integrating all available resources and interventions. The key findings will be helpful in guiding practice, policy development, and future research activities. In the way of optimizing the strategic framework, evaluation of shifting TB epidemiology, risk factors, impacts and cost-effectiveness of interventions, adopting accurate and rapid diagnostic tools, shorter and less toxic preventive therapy are crucial issues. In order to realize targets of End TB strategy, a TB control program for the elderly should be ready globally and regionally with evidence-informed guideline and effective interventions evaluated under particular circumstance.

## Additional files

Additional file 1: Multilingual abstracts in the six official working languages of the United Nations. (PDF 675 kb)

Additional file 2: Descriptive content of the 19 selected articles. (DOCX 29 kb)

## Abbreviations

ACF: Active case-finding
BCG: Bacillus Calmette-Guerin
CDC: US Center for Disease Control
CXR: Chest X-ray
DM: Diabetes mellitus
DOTS: Directly observed treatment, short-course
HIV: Human immunodeficiency virus
IGRA: Interferon-gamma release assay
IPT: Isoniazid preventive therapy
LTBI: Latent TB infection
MDR-TB: Multidrug resistant tuberculosis
MMR: Mass miniature radiography
NTM: Nontuberculous mycobacteria
TB: Tuberculosis
TST: Tuberculin skin test
WHO: World Health Organization
