# Supplementary material for: The strategic framework of tuberculosis control and prevention in the elderly: a scoping review towards End TB targets
Source: Infect Dis Poverty. 2017 Jun 1;6:70. doi: 10.1186/s40249-017-0284-4 (PMC5452345; doi:10.1186/s40249-017-0284-4)
Supplement: Supplementary file 1 — Multilingual abstracts in the six official working languages of the United Nations. (PDF 675 kb) [file 40249_2017_284_MOESM1_ESM.pdf]

Translation of the abstract into the five official working languages of the United Nations

الإطار الاستراتيجي لمكافحة السل والوقاية منه في كبار السن: مراجعة استكشافية نحو أهداف السل النهائية

جون لي، بيوهنوج تشونج، سايروس ل ك لونج، نوبويوكي نيشيكيوري، إميلي ي ي تشان، إنجكيونج يوه

الملخص

مع تسارع وتيرة شيخوخة السكان، تصبح إصابة كبار السن بالسل (TB) تحديا للصحة العامة بشكل متزايد. وعلى الرغم من العبء المتزايد والمخاطر العالية لمكافحة السل في المسنين، فإن الاستراتيجية المستهدفة لم تُفهم وتُقيم جيدا. أجرينا استعراضا استطلاعيا لتحديد استراتيجيات التعامل مع السل الحالية، ثغرات البحوث والسياسات المستهدفة لكبار السن وتلخيص النتائج في إطار استراتيجي نحو تحقيق الأهداف النهائية للتعامل مع السل. كما تم البحث في قواعد البيانات من EMBASE، MEDLINE، Global health، EBM عن الدراسات ، والمقالات، ومراجعات النشر الأصلية التي نشرت باللغة الإنجليزية بين يناير 1990 وديسمبر 2015. أدرجت المواد القائمة على دراسة استراتيجية، أو برنامج، أو المبدأ التوجيهي أو التدخل في كبار السن من منظور الصحة العامة الخاص بالسل.

وافقت تسعة عشر مقالا معايير الإدراج. وكان معظمها من الدراسات النوعية، التي صدرت في البلدان المرتفعة والمتوسطة الدخل، وبعد عام 2000. ولكسر سلسلة انتقال مرض السل وإعادة تنشيط مكافحة العدوى في المسنين، فإن التدخل لتجنب أي تأخير في التشخيص والاحتواء ضروري لمنع انتقال العدوى، وبخاصة في مؤسسات كبار السن والمهاجرين كبار السن؛ فحص السل الكامن والتدابير الوقائية لها آثار فعالة في الحد من خطر إعادة التنشيط، وينبغي أن تستخدم بشكل أقل تحفظ في كبار السن؛ تحسين اكتشاف الحالات مبكرا بوجود مؤشر عال للاشتباه، منهجية الكشف عن المجموعات المعرضة للخطر حسب الأولوية، الاختبارات الأولية والمتابعة المناسبة للعلاج مع الرصد والتقييم، فضلا عن تعزيز إدارة البرامج هي الركائز الأساسية للقضاء على مرض السل النشط. تقييم وبائيات مرض السل، وعوامل الخطر، والآثار والفعالية من حيث التكلفة من التدخلات، واعتماد أدوات التشخيص الدقيقة والسريعة، والعلاج الوقائي الأقصر والأقل سمية، هي من القضايا الهامة لتطوير استراتيجية لدى كبار السن نحو تحقيق أهداف القضاء على السل.

تم تعيين استراتيجيات مكافحة السل في كبار السن بشكل شامل في مسار العلاقة السببية. والإطار والمبادئالتي تم تحديدها في هذه الدراسة تساعد في تقييم وتحسين البرنامج الحالي، ووضع استراتيجية محددة الأهداف، وكذلك جمع فتح النقاشات حول تحديد الأولويات البحثية والتحولات في السياسة المتبعة. ونظرا لندرة السياسات والتدخلات التي تم تقييمها، فضلا عن الجهل بتحول وبائيات واستراتيجية مرض السل خاصة في البلدان النامية، هناك حاجة متزايدة لبرنامج جاهز للسل للمسنين ما يتتبعه إجراء مزيد من البحوث.

Translated from English version into Arabic by Mahmoud Sami, through

TRANSLATORS WITHOUT BORDERS

基于消除结核病目标的老年人结核病防控策略框架：堪域综述

李峻，钟沛康，梁立群，锦织信幸，陈英凝，杨永强

摘要

随着不断加速的人口老龄化，老年人的结核病问题愈发对公共卫生带来挑战。尽管老年人结核病负担不断增加并有更高的发病风险，对其防控策略的了解和评价还十分有限。为明确目前老年人结核病的防控策略、研究和政策缺口，我们开展了一项堪域综述并将结果综合归纳为基于消除结核病目标的策略框架。

在 Embase, MEDLINE, Global health 和 EBM reviews 数据库中，我们搜索了以英文发表于 1990 年 1 月至 2015 年 12 月期间的原创学术研究、文献综述和政策文件，并旨在纳入从公共卫生角度分析老年人结核病防控策略、项目、指南以及干预措施的文章。
19 篇文章符合纳入标准，其中多数文章为定性研究、发表于 2000 年以后和中等及高收入国家。为阻止结核病在老年人中传播和内源性复发的循环发展，感染控制、避免延迟就诊和隔离是预防传播的基础，特别是针对老年人集中居住场所和老龄化的外来移民；对结核病潜伏感染的筛查和预防性治疗可以有效降低内源性复发的风险，应更多的考虑在老年人中实施推广；完善早期患者发现策略，对结核病可疑者高度警惕，在结核病重点高危人群中开展系统筛查，基于经验快速开展早期治疗，密切监控和评估以完成足够的随访治疗，以及强化的项目管理是在老年人中消除活动性结核的主要措施。在制定老年人结核病防控策略时，评价结核病疫情、危险因素以及实施干预措施的影响和成本效果，采用准确和快速的诊断工具、以及缩短的和减少不良反应的预防性治疗方案是实现消除结核病目标的重要手段。
本研究在结核病传播及发病的循环路径中系统地总结了老年人结核病的防控策略。研究提出的框架以及基本准则将有助于评价和完善现有防控项目，开发针对性的防控策略，并且在确定研究优先领域和政策传导方面引起更多的讨论和关注。由于目前尚缺乏相应的政策以及对干预措施的评价研究，特别是在发展中国家对于结核病疫情老龄化以及策略转换的认识尚且不足，急需进一步研究并建立有针对性的防控项目以应对老年人结核病问题。

Translated from English version into Chinese by Jun Li

Le cadre stratégique de contrôle et de prévention de la tuberculose chez les personnes âgées : Étude de cadrage sur les cibles du programme Mettre fin à la tuberculose

Jun Li, Puihong Chung, Cyrus L.K. Leung, Nobuyuki Nishikiori, Emily Y. Y. Chan, Engkiong Yeoh

Résumé

Avec le vieillissement rapide de la population, la tuberculose des personnes âgées devient de plus en plus un défi de santé publique. Malgré la charge croissante et les risques élevés que présente la tuberculose chez les personnes âgées, la stratégie ciblée n’a été ni bien comprise ni évaluée. Nous avons réalisé une étude de cadrage pour recenser les stratégies actuelles de lutte contre la tuberculose, les lacunes de la recherche et des politiques concernant les personnes âgées, et nous en avons récapitulé les résultats dans un cadre stratégique sur les cibles du programme Mettre fin à la tuberculose. Nous avons consulté les bases de données *Embase, MEDLINE*, *Global Health* et *EBM reviews* pour en extraire des études originales, des articles de synthèse et des documents d’orientation publiés en anglais entre janvier 1990 et décembre 2015. Nous avons inclus les articles étudiant les stratégies, les programmes, les directives ou les interventions concernant la lutte contre la tuberculose des personnes âgées du point de vue de la santé publique.
Dix-neuf articles correspondaient aux critères d’inclusion. La plupart étaient des études qualitatives, publiées après 2000 dans des pays à revenu élevé et intermédiaire. Pour rompre le cycle de transmission et réactivation de la tuberculose chez les personnes âgées, il est essentiel de mettre en place la lutte contre l’infection, les interventions destinées à éviter les retards de diagnostic, et le confinement, afin de prévenir la transmission, surtout dans les institutions accueillant des personnes âgées et parmi les immigrants âgés; le dépistage des

infections tuberculeuses latentes et la thérapie préventive ayant des effets marqués sur la réduction du risque de réactivation, ils sont à utiliser avec moins de réticence chez les personnes âgées; les piliers essentiels, pour une élimination active de la tuberculose, sont: l'optimisation de la détection des cas peu avancés lorsque l'indice de suspicion est élevé ; la détection systématique dans les groupes à haut risque prioritaires; le traitement initial empirique, et un traitement de suivi suffisant assorti de contrôles et d'évaluations approfondis ; et une gestion de programme améliorée. Les aspects critiques en vue de la mise au point d'une stratégie visant les cibles du programme Mettre fin à la tuberculose chez les personnes âgées sont l'évaluation de l'épidémiologie tuberculeuse, des facteurs de risque, des effets et du rapport coût-efficacité des interventions, l'adoption d'outils de diagnostic exacts et rapides, et des thérapies préventives moins longues et moins toxiques.
Les stratégies de lutte contre la tuberculose chez les personnes âgées ont été cartographiées globalement sous la forme d'un parcours de liens de causalité. Le cadre et les points principaux mis en évidence dans la présente étude aideront à évaluer et perfectionner le programme actuel, à définir une stratégie ciblée, et à susciter plus de débats sur la façon de fixer les priorités de recherche et les transitions de politiques. Comme il y a très peu d'interventions de politiques et d'évaluations de ces dernières, et que l'évolution de l'épidémiologie tuberculeuse et des stratégies n'est pas suffisamment connue, particulièrement dans les pays en développement, il faut des recherches plus poussées sur la définition rapide d'un programme de lutte antituberculeuse chez les personnes âgées, dont la nécessité se fait de plus en plus sentir.

Translated from English version into French by sinany, through

TRANSLATORS
WITHOUT BORDERS

Стратегические рамки борьбы и профилактики туберкулеза у пожилых людей: анализ мероприятий, направленных на достижение конечных целей

Джун Ли, Пуйхонг Чунг, Сирус Л.К. Лойнг, Нобуюки Нишикиори, Эмили И.И. Чан, Энгкион Йео

Аннотация

Учитывая высокие темпы старения населения, заболеваемость пожилых людей туберкулезом становится все более острой проблемой общественного здравоохранения. Несмотря на растущее бремя и высокие риски заболеваемости туберкулезом для лиц пожилого возраста, не был проведен анализ и оценка целенаправленной стратегии. Мы провели анализ мероприятий с целью выявления существующих стратегий борьбы с туберкулезом, научно-исследовательских и политических пробелов при решении проблем пожилых людей и обобщили полученные результаты в рамках стратегии достижения конечных целей борьбы с туберкулезом. Базы данных Embase, MEDLINE, информационные обзоры по вопросам глобального здравоохранения и научно-доказательной медицине были изучены на предмет наличия оригинальных исследований, аналитических статей и политических документов, опубликованных на английском языке в период с января 1990 по декабрь 2015 гг. Были включены статьи, посвященные

стратегии, программе, руководству или мероприятиям по борьбе с туберкулезом у пожилых людей, с точки зрения общественного здравоохранения.
Девятнадцать статей соответствовали критериям включения. Большинство из них были представлены качественными исследованиями, проведенными в странах с высоким и средним уровнем дохода после 2000 года. Для того чтобы разорвать цепь передачи и реактивации туберкулеза у пожилых людей, важно проводить мероприятия по инфекционному контролю, избежанию задержки в диагностике и сдерживанию распространения заболевания в целях предотвращения его передачи, особенно в учреждениях, предназначенных для пожилых людей и иммигрантов пожилого возраста; скрининг латентной туберкулезной инфекции и профилактическая терапия оказали эффективное воздействие на снижение риска реактивации и должны чаще использоваться для лечения пожилых людей; оптимизации раннего выявление случаев заболевания с высоким индексом подозрения, систематический скрининг для приоритетных групп высокого риска, начальное эмпирическое и адекватное последующее лечение, включая тщательный мониторинг и оценку, а также более эффективное программное управление являются фундаментальной основой ликвидации активного туберкулеза. Оценка эпидемиологии туберкулеза, факторов риска, воздействия и экономической эффективности проводимых мероприятий, внедрение точных и быстрых диагностических инструментов, более короткая и менее токсичная профилактическая терапия являются критически важными аспектами при разработке стратегии лечения пожилых людей при достижении целевых показателей борьбы с туберкулезом.
Стратегии борьбы с туберкулезом у пожилых людей были всесторонне отображены в схеме представления причинно-следственной связи. Структура и принципы, выявленные в данном исследовании, помогут оценить и улучшить текущую программу, разработать целенаправленную стратегию, а также инициировать дополнительные обсуждения на тему приоритетных параметров исследований и изменения политики. С учетом скудости политики и оцениваемых мероприятий, а также неосведомленности об изменений в сфере эпидемиологии и стратегии борьбы с туберкулезом, особенно в развивающихся странах, возрастающая потребность в готовой программе борьбы с туберкулезом для пожилых граждан заслуживает дальнейшего исследования.

Translated from English version into Russian by Irina Zayonchkovskaya, through

TRANSLATORS
WITHOUT BORDERS

El Marco estratégico de control y prevención de la tuberculosis en el anciano: Una revisión bibliográfica en pro de cumplir los objetivos del Fin de la tuberculosis

Jun Li, Puihong Chung, Cyrus LK Leung, Nobuyuki Nishikiori, Emily Y. Y. Chan y Engkiong Yeoh

Resumen

Dado el rápido aumento en el número de la población que llega a una edad más avanzada, la tuberculosis (TB) en los ancianos cada vez más se convierte en un desafío para la salud pública. A pesar del aumento de la carga y del alto riesgo de la tuberculosis en los ancianos, aún no se entiende bien la estrategia enfocada ni tampoco se la evalúa. Reealizamos una revisión bibliográfica para identificar las lagunas en las estrategias, en la investigación y en las políticas para los ancianos con respecto a la TB y se resumieron los resultados dentro de un marco estratégico en pro de los objetivos para el Fin de la TB. Se investigaron las bases de datos de Embase, MEDLINE, Global Health y las revisiones de la MBE en busca de los estudios originales, los artículos de revisión y los documentos de políticas publicados en inglés entre enero de 1990 y diciembre del 2015. Se incluyeron artículos que analizan, desde la perspectiva de la salud pública, la estrategia, el programa, la directriz o la intervención sobre la TB para las personas mayores.

Diecinueve artículos cumplieron con los criterios de inclusión. La mayoría de ellos eran estudios cualitativos hechos en países de ingresos altos y medianos después del 2000. Para romper la cadena de transmisión de la tuberculosis y su reactivación en las personas mayores, es esencial controlar la infección, intervenir para evitar la demora en el diagnóstico y contener, en especial, en las instituciones para personas mayores y en los inmigrantes de edad avanzada. Detectar la infección latente de tuberculosis y la terapia preventiva tuvieron un impacto eficaz en reducir el riesgo de reactivación y deben emplearse con menos reticencia en las personas mayores. Los siguientes son los pilares fundamentales para eliminar la tuberculosis activa: optimizar el hallazgo temprano de casos con un alto índice de sospecha, evaluar sistemáticamente los grupos de alto riesgo prioritarios, hacer un tratamiento empírico inicial y un tratamiento de seguimiento apropiado con un control y una evaluación rigurosos, así como también mejorar la gestión programática. Las siguientes son cuestiones fundamentales para desarrollar una estrategia para los ancianos en pro de los objetivos para la estrategia Fin de la TB: evaluar la epidemiología de la tuberculosis, los factores de riesgo, el impacto y la relación costo-efectividad de las intervenciones, adoptar herramientas de diagnóstico rápidas y precisas, así como también un tratamiento preventivo más corto y menos tóxico.

Las estrategias para el control de la tuberculosis en las personas de edad avanzada fueron mapeadas en forma completa en una ruta de relaciones causales. El marco y los principios identificados en este estudio ayudarán a evaluar el programa actual y mejorarlo, a desarrollar una estrategia enfocada, así como también a plantear más debates sobre establecer prioridades para la investigación y las transiciones en las políticas. La creciente necesidad de un programa preparado contra la tuberculosis justifica una investigación más extensa, dada la escasez de políticas y de las intervenciones evaluadas, así como la falta de conciencia en el cambio de la epidemiología de la tuberculosis y en la estrategia, sobre todo en los países en desarrollo.

Translated from English version into Spanish by LidiaN, through

TRANSLATORS
WITHOUT BORDERS
