# Supplementary material for: The strategic framework of tuberculosis control and prevention in the elderly: a scoping review towards End TB targets
Source: Infect Dis Poverty. 2017 Jun 1;6:70. doi: 10.1186/s40249-017-0284-4 (PMC5452345; doi:10.1186/s40249-017-0284-4)
Supplement: Supplementary file 2 — Descriptive content of the 19 selected articles. (DOCX 29 kb) [file 40249_2017_284_MOESM2_ESM.docx]

**Additional file 1 Descriptive content of the 19 selected articles**

| **Author**  **(country; year)** | **Study object** | **Study aim** | **Study design and method** | **Strategy** | **Key findings and conclusions** |
| --- | --- | --- | --- | --- | --- |
| **Huynh et al. (USA, 2015)** | Elders > 65 years old in China | Explore the impacts of strategies towards End TB targets | Quantitative: an individual-based computational model | ACF combining with the annual health screening; screening and preventive therapy for LTBI | Maintenance of DOTS strategy and all feasible interventions for new transmission had limited impacts. Additional ACF and preventive therapy that limit reactivation would enable China to nearly reach targets. |
| **Zhang, et al. (China, 2015)** | Rural elders ≥ 60 years old in China | Assess feasibility and effects of integrating TB screening within routine health examinations | Quantitative: evaluation of program effectiveness | ACF for the elderly in those with symptoms of TB, patients with type 2 DM, and TB close contacts | ACF in health examinations for TB was effective for the elderly in rural areas, especially among the elderly DM patients with TB symptoms. |
| **Mirsaeidi et al. (USA, 2014)** | NTM infection among the elderly patients | Summarize the characteristics and policy implication | Qualitative: review | Special concerns in nature, diagnosis, treatment, antibiotic therapy, drug interaction, prognosis and management | Comprehensive diagnosis methods and considerable variation in treatment management should be considered for NTM in the elderly. |
| **Verma et al. (Canada, 2013)** | The elderly TB patients on entry to long-term care facilities in Canada | Evaluate the cost-effectiveness of three screening strategy | Quantitative: cost-effectiveness study | Screening for LTBI using the TST and screening for active disease with CXR compared with no screening | Although TST was found more cost-effective than CXR screening, TB screening strategies on entry to long-term care are costly, with large numbers needed to screen. Higher risk of reactivation of LTBI is associated with cost-effectiveness of screening. |
| **Mori et al. (Japan, 2010)** | TB in global ageing population | Explore the epidemiology, risk factors and prevention strategies in ageing population | Qualitative: review | Screening and preventive treatment for LTBI; modification of host factors; intervention in the elderly institutions; clinical concerns about atypical presentation and side effects | Diagnostic tests with better predictive values and shorter, more effective, and less toxic treatment regimens for LTBI and active TB are needed. Until these become available, a high index of suspicion and a careful, holistic approach will continue to be the significant strategy. |
| **Kowada et al.**  **(Japan, 2010)** | BCG-vaccinated, immunocompetent the elderly population (65 years old) in Japan | Evaluate the cost-effectiveness of different screening strategies | Quantitative: cost-effectiveness study | Screening for LTBI/TB using IGRA and screening for TB using CXR compared with no screening | Providing no routine TB screening is currently the most cost-saving strategy, while little role was found for CXR screening. IGRA is the most cost effective assuming high TB/LTBI prevalence. |
| **Schaaf et al. (South Africa, 2010)** | The elderly (>65 years) | Describes public health aspects of TB control at extremes of age | Qualitative: review | Prevent transmission of infection; identify LTBI and preventive therapy; early diagnosis and timely initiation of treatment | Appropriate investigations and prompt treatment, especially screening for high-risk groups and prevention in institutional care, will help to decrease morbidity and mortality. |
| **Van Den Brande. (Belgium, 2006)** | The elderly | Explore the prevention and management of TB and LTBI in the elderly patient | Qualitative: review/guideline | Early diagnosis, empirical initiation and follow-up treatment; preventive treatment for LTBI; prevent transmission especially in nursing homes | Treatment in the elderly does not essentially differ from that in younger age groups. Earlier diagnosis by screening LTBI and TB, initial and follow-up during treatment, treatment for LTBI and institutional care should be used less reluctantly in the elderly. |
| **Van Den Brande.**  **(Belgium, 2005)** |  |  |  |  |  |
| **Ghotbi et al. (Japan, 2005)** | TB control priority for the elderly (>65 years) in Japan | Study national TB control strategy and priority with cost-effectiveness consideration | Qualitative: review | Effective strategies of both active and passive case finding in the elderly through public and community health services | TB control measures should be targeted to the elderly people. Public health programs for passive case finding, DOTS strategy management, and pilots on active community case-finding programs were needed. |
| **Thrupp, L. et al. (USA, 2004)** | The elderly in long-term care facilities | Provide guideline to assist healthcare providers for TB control and prevention | Qualitative: review/guideline | Diagnosis, treatment and management of LTBI and TB patients, infection control. | Rapid recognition of possible disease, diagnosis, treatment and implementation of airborne precautions were essential to prevent transmission. |
| **Zevallos et al. (USA, 2003)** | The elderly in USA | Describe the core parts and main challenges in TB control strategy | Qualitative: review | The evaluation of TST, preventive therapy, institutional care and treatment management. | TST should be targeted at persons with risk factors for developing TB; isoniazid is the best method of preventing LTBI from activation; all nursing home residents must be regularly screened for LTBI; empiric TB treatment should be more ready. |
| **Rajagopalan et al. (USA, 2000)** | The elderly in long-term care facilities | Guide the TB control strategy in long-term care facilities | Qualitative: review | Surveillance, diagnosis, containment, treatment and prevention, assessment and education | The institutionalized the elderly were at a greater risk for both reactivation of LTBI and new infection. Comprehensive guidance should be emphasized for infection control in long-term care facilities. |
| **Rajagopalan et al. (USA, 2000)** | The elderly | Discuss TB diagnosis, treatment, prevention and management | Qualitative: review/guideline | TST, preventive therapy, treatment assessments and monitoring, infection control. | TST and more aggressive diagnostic intervention are needed. Preventive therapy, treatment assessment and monitoring, and infection control are essential for the elderly. |
| **Rajagopalan et al. (USA, 2001)** |  |  |  |  |  |
| **Marchand et al. (Canada, 1999)** | The elderly in long-term care facilities in Canada | Examine the cost-effectiveness of active LTBI screening and chemoprophylaxis | Quantitative: cost-effectiveness study | Systematic screening the elderly patients newly admitted to facilities by TST comparing to passive strategy | Screening with the TST and chemoprophylaxis for high-risk reactors was more cost-effective than current services widely used. |
| **Gubser (USA, 1998)** | The elderly in USA | Discuss implications of TB prevention in community and nursing home settings | Qualitative: review | Test by using TST, treatment for active cases, preventive therapy and education | Special concerns on the elderly TB clinical manifestation, test, treatment, prevention and education were essential for healthcare provider in community or nursing home settings. |
| **Walsh (USA, 1994)** | The elderly | Provide guidelines on recommendations for TB prevention and control | Qualitative: review/ guideline | Guideline and explanation for all aspects of care, the role of the primary practitioner, current diagnostic testing and recommended treatment for TB. | Primary providers placed an important role of TB prevention and education. Promote routine screening; preventive therapy and effective treatment must be given for both institutionalized and community-based the elderly. |
| **CDC (USA, 1990)** | The elderly in long-term care in USA | Describe TB control activities | Qualitative: guideline  /recommendation | Surveillance, containment, assessment and education | Long-term care facilities and health department should take responsibilities and implement these standardized measures. |
